# A Perspective on Thiazolidinone Scaffold Development as a New Therapeutic Strategy for Toxoplasmosis

**DOI:** 10.3389/fcimb.2018.00360

**Published:** 2018-10-16

**Authors:** Cristian Rocha-Roa, Diego Molina, Néstor Cardona

**Affiliations:** ^1^Centre for Biomedical Research CIBM, University of Quindío, Armenia, Colombia; ^2^Dentistry Faculty, University Antonio Nariño, Armenia, Colombia

**Keywords:** Thiazolidinone scaffold, new drug, *Toxoplasma gondii*, toxoplasmosis, *in silico*

## Abstract

*Toxoplasma gondii* is one of the most successful parasites due to its ability to infect a wide variety of warm-blooded animals. It is estimated that one-third of the world's population is latently infected. The generic therapy for toxoplasmosis has been a combination of antifolates such as pyrimethamine or trimethoprim with either sulfadiazine or antibiotics such as clindamycin with a combination with leucovorin to prevent hematologic toxicity. This therapy shows limitations such as drug intolerance, low bioavailability or drug resistance by the parasite. There is a need for the development of new molecules with the capacity to block any stage of the parasite's life cycle in humans or in a different type of hosts. Heterocyclic compounds are promissory drugs due to its reported biological activity; for this reason, thiazolidinone and its derivatives are presented as a new alternative not only for its inhibitory activity against the parasite but also for its high selectivity-level with high therapeutic index. Thiazolidinones are an important scaffold known to be associated with anticancer, antibacterial, antifungal, antiviral, antioxidant, and antidiabetic activities. The molecule possesses an imidazole ring that has been described as an antiprotozoal agent with antiparasitic properties and less toxicity. Thiazolidinone derivatives have been reportedly as building blocks in organic chemistry and as scaffolds for drug discovery. Here we present a perspective of how structural modifications of the thiazolidinone core could generate new compounds with high anti-parasitic effect and less toxic results.

## Introduction

To date, pyrimethamine and sulfadiazine with corticosteroids continue to be the gold standard in treating toxoplasmosis (Jasper et al., 2013); this therapy has presented reported cytotoxicity that is minimized using leucovorin, nevertheless, hematologic toxicity is still a problem to overcome. Literature reports show that in encephalitis caused by *Toxoplasma*, 62% of patients presented toxicity and severe side effects (Porter and Sande, 1992), or discontinuation of pyrimethamine-sulfadiazine in a group of patients (Dannemann et al., 1992; Katlama et al., 1996). When cases of allergy to sulfa drugs appear, a replacement with clindamycin can be applied but the efficacy is lower and the toxicity is similar (Katlama et al., 1996). As an alternative, trimethoprim-sulfamethoxazole shows similar effect to pyrimethamine-sulfadiazine (Alday and Doggett, 2017). For ocular toxoplasmosis, for example, the most frequent chemotherapeutic regime consists of pyrimethamine-sulfadiazine plus corticosteroids; this classical approach may have some risks that depend on patient susceptibility to drug toxicity or allergic reactions (Park and Nam, 2013). Other alternative therapy options are based on the use of atovaquone or azithromycin in combination with pyrimethamine or sulfadiazine but supported by less clinical data and similar rates of patient intolerance (Alday and Doggett, 2017). In general terms, evidence supports the idea that the actual drug regimen used to treat toxoplasmosis could be improved, and the fact that toxicity is the major problem reported strengthens the idea that exploring new pharmaceutical alternatives that would improve the care of patients is feasible.

## *Toxoplasma* and toxoplasmosis

*Toxoplasma gondii* is an obligate intracellular protozoan parasite that belongs to the phylum Apicomplexa, it has the ability to infect blood all warmed animals all around the world. Other Apicomplexans medically important such as *Cryptosporidium, Babesia*, and *Plasmodium* share biological similarities that make them susceptible to antifolate drugs i.e., pyrimethamine and sulfonamides (Alday and Doggett, 2017). It is estimated that about one-third of the world's population is latently infected with *T. gondii*. The seroprevalence of infection with *T. gondii* is influenced by cultural, hygienic, and nutritional habits, and by climate and environmental conditions (Sroka et al., 2010); with prevalence rates of infection among healthy people ranging from 7.5 to 80% worldwide (Peng et al., 2015).

*Toxoplasma gondii* presents a complex life cycle with a variety of intermediate hosts. The parasite enters the human mainly by four routes of infection: (i) by consumption of raw or undercooked meat containing viable tissue cysts, (ii) eating food products or drinking water contaminated with oocysts, (iii) transmission of tachyzoites to the fetus through the placenta and (iv) organ transplantation (Peng et al., 2015) or blood transfusion (Alvarado-Esquivel et al., 2018). When acquired via oral, bradyzoites and sporozoites are released from cysts and oocysts invading intestinal cells, thereafter, tachyzoites are differentiated which is the parasite-disseminated form that travels via the blood or lymphatic system to different anatomic regions inducing an acute or chronic infection (Wohlfert et al., 2017).

Host cell invasion is an important event in which three parasite's organelles are mainly involved: micronemes, rhoptries (composed by two different substructures: rhoptry neck and rhoptry bulb) and dense granules. Proteins secreted by these three organelles are crucial in host cell attachment, penetration, and in the formation of the parasitophorous vacuole. Micronemes proteins (MICs) are involved in attachment to the host's membrane receptors. Rhoptries neck proteins (RONs) are released following micronemes to form the moving junction, this structure is important to form the parasitophorus vacuole membrane (PVM) using the host's membrane but without proteins. Then, rhoptries bulb proteins (ROPs) are released within vacuoles to the cytosolic face of PVM. Finally, dense granules proteins (GRAs) are released into the PVM after invasion (for a complete understanding of MICs, RONs, ROPs, and GRAs function please review J. Laliberté and V. B. Carruthers) (Laliberté and Carruthers, 2008). There are reports that show the importance of proteins involved like ROP18, ROP5, ROP16, and GRA15 in immune modulation depending on the strain type, such as prolonged STAT3/6 activation, reduced IL-12 production, and avoidance of parasite clearance which are related to high virulence in type I strains; non-sustained STAT3/6 activation, high levels of IL-12 and enhanced parasite clearance related to intermediate virulence in type II strains; and prolonged STAT3/6 activation, reduced IL-12 production and enhanced parasite clearance related to low virulence in type III strains (Hunter and Sibley, 2012).

The importance of knowing the bulk of proteins that plays a role as virulence factors or implicated in the host cell invasion process is to have a broad panorama to select potential new drug targets.

Infection with *T. gondii* brings clinical complications such as ocular, neurological and systemic disease mainly in immunocompromised patients and those infected congenitally. Ocular toxoplasmosis is one of the most common causes of posterior uveitis in 20–60% of cases and, in some countries it is one of the most important causes of visual impairment (de-la-Torre et al., 2014). Neurological complications are characteristic of an acquired immunodeficiency syndrome (AIDS) and are one of the causes of CNS mass lesions in AIDS. Cerebral toxoplasmosis is also associated with high mortality and morbidity in patients with states of immunocompromised (Patil et al., 2011). In congenital toxoplasmosis, the infection is acquired during pregnancy and can have devastating consequences in the fetus; in some cases, the infection develops an ocular form that can reactivate depending on different factors (Wallon and Peyron, 2018).

## Thiazolidinones core on *Toxoplasma gondii* and drug alternatives

Due to the biological properties of thiazolidinones, this pharmacologic core appears as a good alternative against toxoplasmosis. One of the forms for obtaining the thiazolidinone core arises after the combination of two precursors of which important biological activities are known, hydroxyurea and thiosemicarbazone (Tenório et al., 2005). Hydroxyurea have shown a strong effect on the intracellular elimination of protozoa such as *T. gondii, T. cruzi*, and *L. amazonensis* (de Melo et al., 2000). On the other hand, it has been reported that thiosemicarbazones are potential inhibitors of the ribonucleotide reductase enzyme, which is responsible of deoxyribonucleotides synthesis (Liu et al., 1992). The activity of thiosemicarbazones is related to the capacity to chelate metal atoms that are important for the survival of the parasite; this characteristic is also shared with the group arylhydrazone, moiety that has been used for the improvement of the biological activity of the thiazolidinone core (Walcourt et al., 2004). From another point of view, it could be thought that the fact of gathering structural cores with important biological activities is one of the factors that give the thiazolidinone compounds a broad spectrum of pharmacological activity; for this reason, thiazolidinone core derivatives have become object of study due to its numerous biological activities, becoming a promissory scaffold with pharmaceutical potential and with anti-*Toxoplasma* effects (Kaur Manjal et al., 2017).

The first study to link thiazolidinone compounds with anti-*Toxoplasma* activity was reported by Tenório et al. (2005), who designed a series of substituted thiosemicarbazone compounds in the arylhydrazone moiety with nitro substituents in the *ortho, meta* and *para* positions; they also designed a series of thiazolidinones substituted on the nitrogen atom of the 3-position with phenyl, methyl, ethyl and hydrogen groups, in addition, nitrobenzene groups were substituted on the moiety arylhydrazone that is attached to the carbon of the 2-position (Tenório et al., 2005). The 2-position has been described for a long time as one of the most studied and promising positions for the design of drugs based on the thiazolidinone heterocycle (Hamama et al., 2008). In addition, reports of a substitution of the carbon at 5-position for an acetic acid group in the thiazolidinone heterocycle have been made. After *in vitro* experiments, it has been shown that the thiazolidinone derivatives were more efficient on the intracellular elimination of *T. gondii* than the thiosemicarbazone derivatives and hydroxyurea (reference drug), resulting in a lower percentage of infected host cells; the treatment with thiazolidinones resulted in up to 4 intracellular parasites, whereas thiosemicarbazones and hydroxyurea treatment resulted in up to 72 and 186 intracellular parasites, respectively (Tenório et al., 2005). In 2008, de Aquino et al. (2008) continued with the scaffold designed by Tenório et al. (2005); they kept the phenyl group on 3-position, the arylhydrazone group on 2-position and the acetic acid group on 5-position of the thiazolidinone heterocycle. At the same time, they evaluated compounds resulting from the addition of phenyl groups on positions 3 and 4 of the thiosemicarbazone core on the aromatic rings of the arylhydrazone moieties of thiazolidinones and thiosemicarbazone; additions of electron-withdrawing or electron-donating radicals were also evaluated. These modifications resulted in thiazolidinone derivatives that had an effective action on intracellular parasite multiplication, as a consequence, the mean number of normal tachyzoites decreased. The concentration ≤ 0.1 mM of some thiazolidinone derivatives resulted in a 50% inhibition of parasite growth; this concentration is represented in a range of 12.5–30 μg/mL. In contrast, effective sulfadiazine concentration in the same *in vitro* conditions was 3 mM. In conclusion, some thiazolidinones reported in this work resulted to be more effective than hydroxyurea at 0.5 mM concentration (de Aquino et al., 2008).

More data of a new series of thiazolidinones and thiosemicarbazones was reported by Carvalho et al. (2010). They kept the arylhydrazone group on the thiosemicarbazones and thiazolidinones and they did not retain the aromatic ring on 3-position of the thiazolidinone core. On the phenyl group of the moiety arylhydrazone they made additions of hydrogen, chlorine, and nitro in the *para* position. The best molecule derivatives were able to drastically decrease the average number of intracellular parasites, effects that are very promising compared to the pharmacological treatments currently used. In addition, these authors suggest some morphologically effects caused to the intracellular parasites, including the development of a process of vesiculation in the cytoplasm of the *T. gondii* ending up in altering the parasite's cell cycle. This is a first approach to the possible effects of this type of compounds on *T. gondii* (Carvalho et al., 2010).

On the other hand, compounds such as benzinidazole, miconazole, ketoconazole, metronidazole, and others, are currently widely used as therapeutic agents; these have in common the presence of a heterocyclic imidazole ring in its structure, adding up to an extensive list of studies of derivatives with powerful biological activity (Zhang et al., 2014). For this reason, Liesen et al. (2010) mixed the imidazole ring with the core thiosemicarbazide, thiazolidinone, and thiadiazole. In the case of thiazolidinones, the imidazole ring was bound by the region of the hydrazone group. These new compounds were evaluated in Vero cells infected with tachyzoites of *T. gondii*, showing elimination of parasites; the most active compounds were thiosemicarbazide and thiadiazole derivatives at 0.1 mM concentration, while thiazolidinone derivatives showed anti-*Toxoplasma* activity only at 1 mM concentration. Although, the compounds that showed activity on the elimination of intracellular parasites with concentrations of 1 mM are not the best, they can be taken as starting point for further studies in order to improve their activity and reach effective concentrations in the scale of μM and even nM. In this study the majority of the compounds presented high toxicity, and all the compounds evaluated showed drastic changes in the morphology of the parasite as the incubation time passed and also a better activity in comparison with the standard drugs sulfadiazine and hydroxyurea at 10 mM concentration (Liesen et al., 2010).

In another work, Aquino et al. (2011) evaluated the addition of 4-nitrobenzylidene as a new group located in the carbon of 5-position of the thiazolidinone heterocycle and retained the moiety arylhydrazone, in which some substitutions were made in order to explore new pharmacological alternatives with promising anti-proliferative effect of *T. gondii* cultivated *in vitro*; resulting in new thiazolidinone derivatives that showed elimination of parasites in Vero cells at 0.02–0.7 mM concentration and a Mean Lethal Dose (LD_50_) at >10 mM. These derivatives were most effective compared with the reference drugs hydroxyurea and sulfadiazine, which showed LD_50_ at 1 mM and 8 mM, respectively. Authors report that after drug treatment the tachyzoites showed big morphologic damages prior to complete elimination (Aquino et al., 2011). Alternatively, D'Ascenzio et al. reported a different evaluation in 2014. They evaluated two series of molecules with a total of 74 new thiazolidinone derivatives. The synthesis consisted of the addition of linear, branched, cyclic and heterocyclic carbonyl groups on the hydrazonic nitrogen-1 (moiety coupled to the thiazolidinone core at 2-position). The main difference between the two series was a substitution for a benzyl ring on the nitrogen at 3-position of the thiazolidinone core. The data reported by D'Ascenzio shows therapeutic index values (TI) for thiazolidinone derivatives against *T. gondii*; each compound was tested for anti-*Toxoplasma* as well as for cytotoxic activity in HFF (human foreskin fibroblast) resulting in compounds with effective concentrations in the micromolar scale (≤ 10 μM), equalling and even overcoming the effect and cytotoxicity levels of the control drug trimethoprim. In addition, some of these compounds were able to decrease the attachment and invasion of tachyzoites to the host cells, suggesting an extracellular effect with potent anti-parasitic activity (D'Ascenzio et al., 2014).

In an effort to find new compounds with better effect against *T. gondii* using computational tools, Asadollahi and Mani in 2015 performed a predictive model of Quantitative-Structure-Activity-Relationship (QSAR) using 68 of the molecules reported by D'Ascenzio et al. (2014). The obtained QSAR model was successfully trained for the prediction of therapeutic index values for the new thiazolidinone derivatives used against *T. gondii*. The authors suggest that this model can be used as a complementary tool in the search for new therapeutic agents with anti-*Toxoplasma* activity (Asadollahi-Baboli and Mani-Varnosfaderani, 2015).

**Figure 1:**
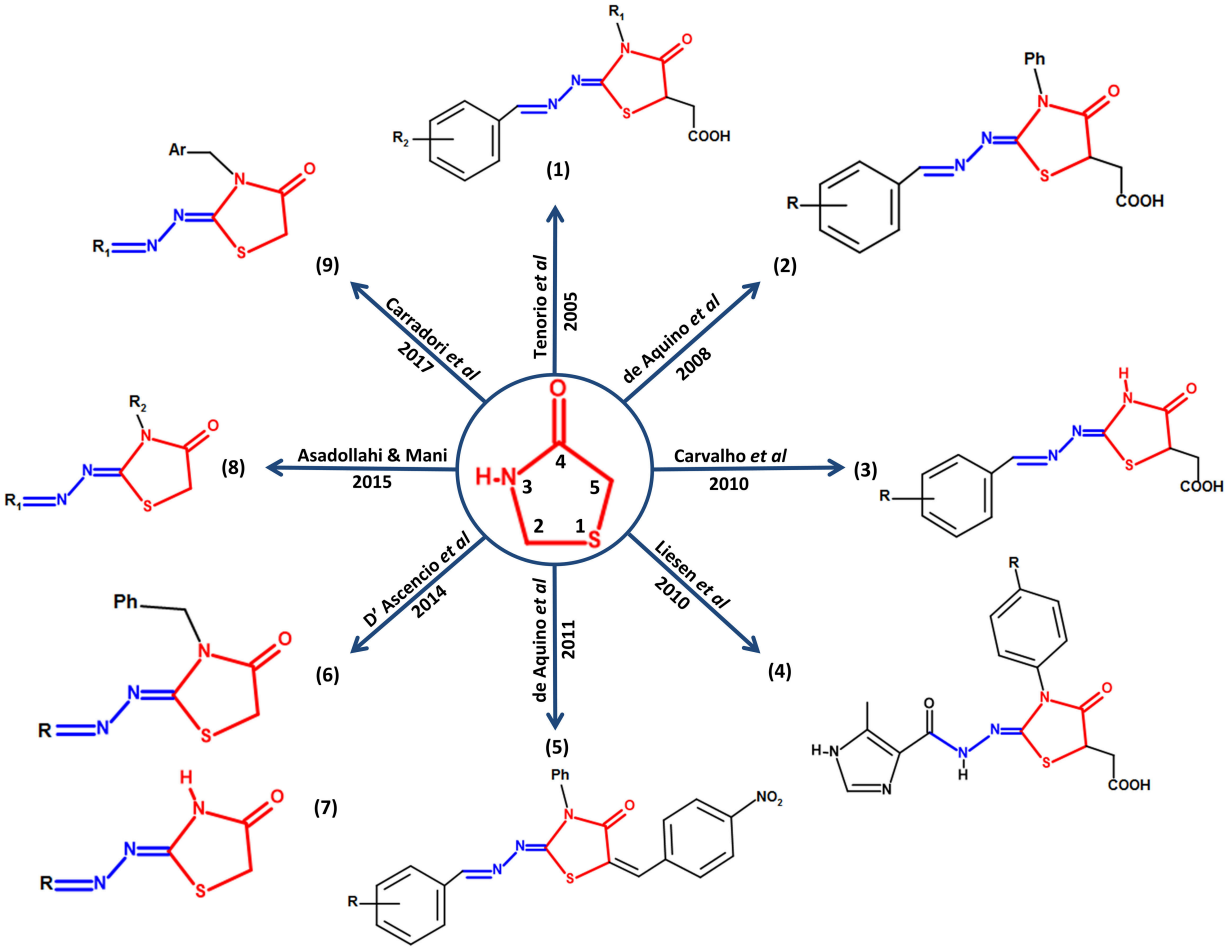
Structural modifications performed on the thiazolidinone core as an alternative for the search of new drugs with possible anti-*Toxoplasma* activity. In the center of the figure, the thiazolidinone core is shown in red with its respective numbering and its conservation around it in all the studies in which they report activity of thiazolidinone derivatives against *T. gondii*. In addition, the structure of moiety hydrazone conserved in position 2 of the heterocyclic thiazolidinone ring is highlighted in blue.

Carradori et al. (2017) synthesized and evaluated a series of 33 new compounds derivatives, in which they kept the thiazolidinone core and only different substituent groups varied on the lactate nitrogen of the heterocycle and nitrogen-1 of the moiety hydrazone. These compounds showed better effects compared against sulfadiazine *in vitro* in terms of inhibition of growth, invasion, and replication of *T. gondii*. Carradori et al suggest that a substitution with a ferrocene group on nitrogen-1 of the moiety hydrazone could be a candidate modification to enhance the effect of the thiazolidinone core since the compounds that presented this group showed high effectiveness against the host cell invasion and the replication of the parasite, also low cytotoxicity; reaching effective concentrations in the micromolar scale. Some compounds showed better values of Median Toxicity (TD_50_) at ≥320 μM, compared with TD_50_ at 281 μM of the reference drug sulfadiazine. The thiazolidinone derivatives reached lower values of IC_50_ at 5 μM, while the control drug sulfadiazine showed an IC_50_ value of 43 μM. In addition, these new compounds had a promising effect over attached extracellular *T. gondii* tachyzoites to host cell, as well as the compounds described by D'Ascenzio et al. (2014), converting them into encouraging compounds for an alternative therapy against *Toxoplasma* (Carradori et al., 2017). Figure 1 summarizes a timeline of all the modifications reported up to 2017 on the thiazolidinone core against *T. gondii*.

Finally, it has been noted that the thiazolidinone core has become a key candidate for the development of drugs with anti-*Toxoplasma* activity. It was also observed that moiety hydrazone was preserved in all studies, which suggests that this moiety is also part of a possible promising scaffold with antiparasitic activity, and recent studies suggest that the presence of moiety hydrazone on the drug would be potentiating its biological activity (Leite et al., 2017; Vargas et al., 2018). It is worth to mention that there are also numerous studies in which the use thiazolidinone-like structural core against *T. gondii* are reported, for example the thiazole core, for which a broad spectrum of biological activity, including anti-*Toxoplasma*, has also been documented (Chimenti et al., 2009; Hencken et al., 2010; McFarland et al., 2016).

## *In silico* approaches for rational design and development of drugs against *Toxoplasma gondii*

A good start point to describe the mode of binding of drug-like molecules is to analyze structural crystallizations that present derivatives of the thiazolidinone core in complex with proteins of parasitic origin. For instance, in *Plasmodium malariae* crystallographic reports with thiazolidine derivatives includes the crystal structure of an aspartic protease (PDB:2ANL) (Clemente et al., 2006); in *P. falciparum*, 3 crystallized proteins of the plasmepsin family (PDB: 3QS1, 3FNU, and 3QVI) (Bhaumik et al., 2009, 2011a,b); these aspartic proteases have been described as important for the life cycle of the parasite and also as a strategy to decrease the survival and proliferation of *T. gondii* (Li et al., 2012; Zhao et al., 2017). Another example is *Leishmania major* with a N-myristoyltransferase protein (PDB:5AG4) (Spinks et al., 2015). N-myristoyltransferase as well as palmitoyltransferase, which cause post-translational changes, has been described as keys for invasion, motility, cell morphology, and with a possible role in the formation of daughter cells in *T. gondii* (Foe et al., 2015; Caballero et al., 2016; Brown et al., 2017). However, it should be mentioned that by means of X-ray crystallography and NMR spectroscopy, molecular-targets of drug-like molecules could not be identified. For thiazolidinones target identification in parasites techniques such as enzymatic inhibition assay using recombinant proteins and scintillation proximity assay (Xia et al., 2016) has been used; these type of techniques are part of the so-called direct biochemical methods, since they contemplate the direct interaction between the drug and the purified protein. There are also other methodologies such as genetic interaction manipulation, which are based on the suppression or enhanced expression of the gene of the molecular-target in the cell, which allow generating target hypotheses of drug treatment. Finally, there are computational approaches that are of great help in obtaining a robust understanding of ligand-receptor interactions. These three alternatives are complementary to each other and can give an insight of possible pharmacological targets of thiazolidinones compounds against *T. gondii* (Schenone et al., 2013). Regarding computational approaches, some investigations have explored the possible pharmacodynamics of thiazolidinone derivatives in intracellular parasites, for example, the work by Kumar et al. (2010) and Kaushik et al. (2015) in which they studied proteins such as Lactate dehydrogenase and Enoyl-ACP reductase of *P. falciparum*, respectively, and Cruzain of *Trypanosoma cruzi* (Moreira et al., 2014) using molecular docking techniques to elucidate the energy of interaction of ligand-receptor. In *T. gondii*, such proteins could have homologs that are worth the effort to analyze, as the case of Cruzain that have a functional protein, the cysteine protease Cathepsin L (Huang et al., 2009).

It is worth to mention that the discussed above proteins or those implicated in attachment or invasion to the host cell, like ROPs, RONs, MICs, or GRAs may become candidates for new drug-targets in *T. gondii*, even opening the possibility of a multi-target and multistage effect on the parasite, being this a good approximation of the possible pharmacodynamics and one of the explanations of the potent biological activity of thiazolidinone compounds on *T. gondii*. Traditionally, drugs has been designed and directed to interact with a single target, giving them specificity, but due to current incurable pathologies and drug resistance, it has been clearly seen that in some cases a single target is not an effective treatment, to overcome this, a multi-target effect within the same pathogen is a promising alternative to enhance a pharmacological effect (Ramsay et al., 2018; Sestito et al., 2018).

Recent work in our research group using computational experiments as a first approximation of the pharmacodynamics of thiazolidinone compounds reported by other authors on *T. gondii* (D'Ascenzio et al., 2014; Carradori et al., 2017), suggests a possible preference of these derivatives for kinase proteins, such as TgCDPK1 and especially TgROP18, which has been described as unique and crucial for virulence of the parasite; the compounds caused drastic conformational changes increasing the distance between the catalytic residues, suggesting an inactivation of the kinase activity caused by such changes, these analyses were performed using molecular docking and molecular dynamics simulations (unpublished data).

**Figure 2:**
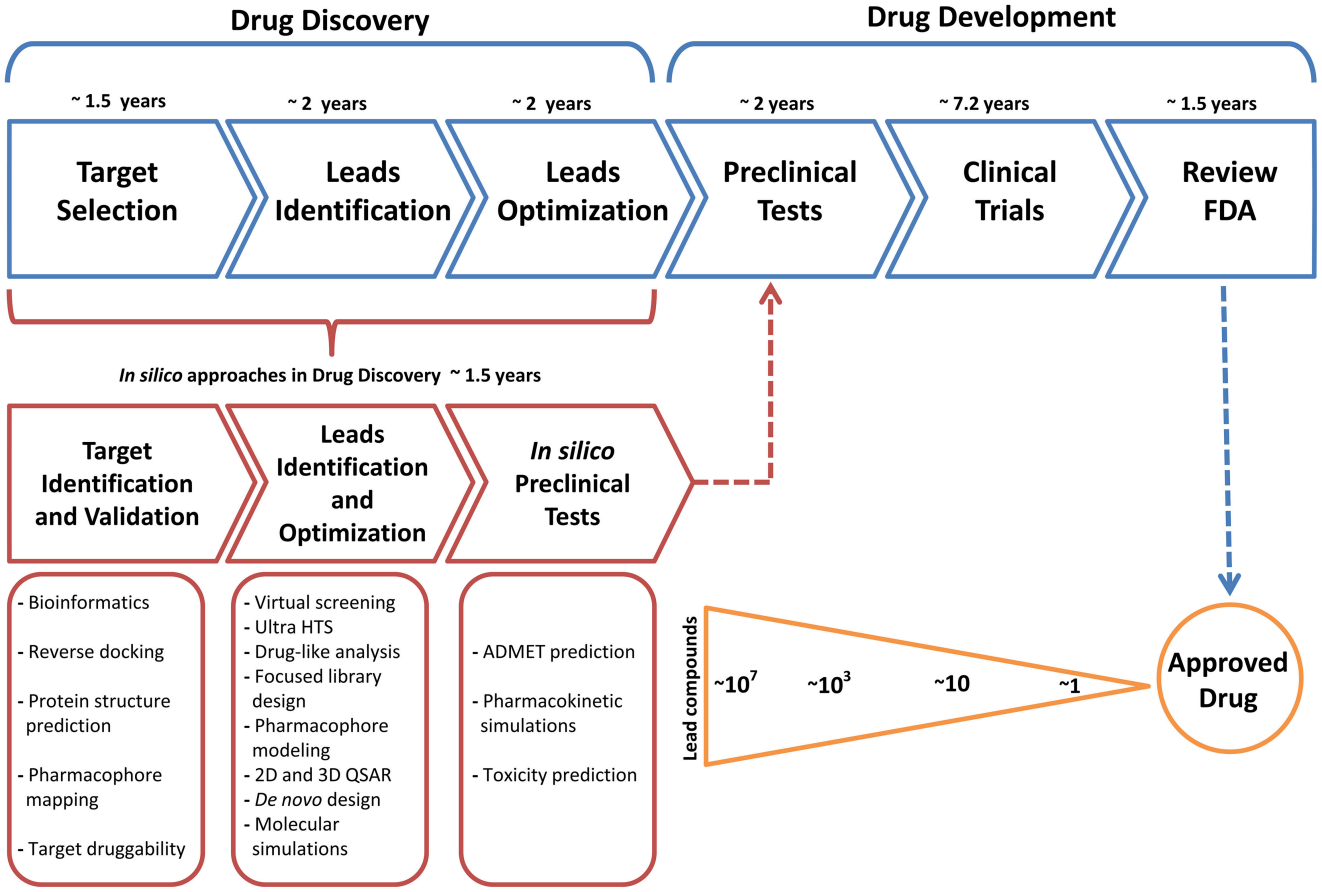
Common timeline for drug discovery/drug development and the key role of the approaches of computational methodologies.

In general terms, the development path for new molecular and biological entities approved by the FDA requires in average 7 years or more from the start of the clinical trials to regulatory approval (Kaitin, 2010); additionally, the process of drug discovery before the drug development takes up to 5 years (Figure 2), this initial process can be time improved in terms of time using computational methods as a complementary tools. In recent years, computational methods have played an important role in efforts to obtain effective compounds that can overcome the limitations of current pharmacological treatments for various diseases. Obtaining these bioactive compounds is based on a rational design, where bioinformatics tools are used to provide crucial information to identify and describe a suitable target. A large number of computational tools available provide support to surpass different kinds of problems, for example, if the structure of a target biomolecule such proteins, DNA or RNA are unknown, it can be obtained through molecular modeling techniques like Homology Modeling, which constructs a three-dimensional model by combining the input information and the experimental structures resolved previously (Khan et al., 2016), generally recorded in the Protein Data Bank (https://www.rcsb.org/) (Berman et al., 2000). It should be mentioned that in some cases the drug discovery follow two routes: (i) drug design based on the structure (when the structural specific site of the target is known) and (ii) drug design based on the ligand (when starting from a compound lead with known effect and the structure of the receptor is unknown). Both routes generally start with an extensive number of lead compounds in order to reduce it to a small group of molecules with the desired effect.

Methodologies such as molecular docking simulations accompanied by a High Throughput Screening, QSAR models are usually used. As the number of compounds decreases, more precise and robust tools have to be used, like Quantum Mechanics (QM) simulations, Molecular Mechanics simulations and Hybrid or multi-scale simulations, as Quantum Mechanics/Molecular Mechanics simulations; these techniques allow us to obtain information in greater detail about the mode of interaction of a ligand or about the general behavior of a biological ligand-receptor complex; for example, QM/MM simulations allow the study of enzyme reaction mechanisms, as well as the understanding of electron and structural features that control the reactivity of the molecules. Another type of simulations are so-called Coarse Grained because its main characteristic is the reduction of the number of particles, allowing to simulate bigger biological systems for much longer times, even in the millisecond scale, unlike All Atom simulations (MM simulations) which usually is in the nanosecond and microsecond scale. For more related information about the Multiscale simulations please review the work of Dans et al. (2016). It should be mentioned that the simulated time scale is directly related to the biological phenomenon of interest, for example, protein folding or unfolding, conformational changes by ligand-receptor interaction or protein attachment to plasmatic membranes, etc. (Ou-Yang et al., 2012; Sliwoski et al., 2013; Mohd Hassan et al., 2014).

Based on all the above, a good option to find effective drugs against *Toxoplasma*, would be to identify one of the many molecular targets of thiazolidinone in *T. gondii* and to use *in silico* approaches to adapt a more specific thiazolidinone derivative that is highly selective for a molecular-target of interest, as in the same mechanism as many kinase inhibitors that work in a specific way despite the similarity among kinases (Ferguson and Gray, 2018).

## Conclusion

The thiazolidinone scaffold has been showed as a promising drug-like compound against *T. gondii* due to its demonstrated biological effects and the experimental information reported by several authors; these type of data facilitates to propose studies with rational design approaches that can result in new pharmacological alternatives using specific molecular targets from the parasite; in addition, a bulk of evidence show that *in silico* approaches linked with experimental work can result in new workflow schemes that favor the process of design and development of new drugs. The tools of modern drug discovery using experimental and *in silico* techniques have the power to analyze millions of compounds to determine their potential association with a single or multiple target would contribute to improve the activity of obsolete compounds or the generation of new ones, allowing for substantially reduced time and costs for the development and discovery of new drugs. Finally, the better understanding of the mechanism of action of the drug can lead to its improvement, granting specificity to the compound and therefore increasing its effectiveness. These methodologies respond to the call of the rational design of drugs against toxoplasmosis and other pathologies.

## Author contributions

DM and NC conceived the idea for the publication; DM, CR-R, and NC prepared the manuscript. All authors read and approved the final manuscript.

### Conflict of interest statement

The authors declare that the research was conducted in the absence of any commercial or financial relationships that could be construed as a potential conflict of interest.
